# Improvement of Salt Tolerance Using Wild Rice Genes

**DOI:** 10.3389/fpls.2017.02269

**Published:** 2018-01-17

**Authors:** Ruidang Quan, Juan Wang, Jian Hui, Haibo Bai, Xuelian Lyu, Yongxing Zhu, Haiwen Zhang, Zhijin Zhang, Shuhua Li, Rongfeng Huang

**Affiliations:** ^1^Biotechnology Research Institute, Chinese Academy of Agricultural Sciences, Beijing, China; ^2^National Key Facility of Crop Gene Resources and Genetic Improvement, Beijing, China; ^3^Ningxia Academy of Agriculture and Forestry Sciences, Yinchuan, China

**Keywords:** wild rice, cultivated rice, salt tolerance, QTL, gene introgression

## Abstract

Salt stress causes significant reductions in rice production worldwide; thus, improving salt tolerance is a promising approach to meet the increasing food demand. Wild rice germplasm is considered a valuable genetic resource for improving rice cultivars. However, information regarding the improvement of salt tolerance in cultivated rice using wild rice genes is limited. In this study, we identified a salt-tolerant line Dongxiang/Ningjing 15 (DJ15) under salt-stress field conditions from the population of a salt tolerant Dongxiang wild rice × a cultivated rice variety Ningjing16 (NJ16). Genomic resequencing analysis of NJ16, DJ15 and Dongxiang wild rice revealed that the introgressed genomic fragments were unevenly distributed over the 12 chromosomes (Chr.) and mainly identified on Chr. 6, 7, 10, and 11. Using quantitative trait locus (QTL) mapping, we found 9 QTL for salt tolerance (qST) at the seedling stage located on Chr. 1, 3, 4, 5, 6, 8, and 10. In addition, sequence variant analysis within the QTL regions demonstrated that SKC1/HKT8/HKT1;5 and HAK6 transporters along with numerous transcriptional factors were the candidate genes for the salt tolerant QTL. The DJ15/Koshihikari recombinant inbred lines that contained both qST1.2 and qST6, two QTL with the highest effect for salt tolerance, were more tolerant than the parental lines under salt-stress field conditions. Furthermore, the qST6 near-isogenic lines with IR29 background were more tolerant than IR29, indicating that qST1.2 and qST6 could improve salt tolerance in rice. Overall, our study indicates that wild rice genes could markedly improve the salt tolerance of cultivated rice.

## Introduction

Rice is one of the most important staple crops worldwide. The global demand for staple crops, including rice, increases with the continuous increase of human population. However, the production of staple crops is threatened by abiotic stresses including salt, drought and high/low temperature (Arzani and Ashraf, 2016; Calanca, 2017). Therefore, improving the productivity of crops in salt-stressed areas is considered essential to meet the increasing food demand.

Compared with wheat, barley and cotton, rice is a more salt sensitive crop (Chinnusamy et al., 2005). Salt tolerance in rice changes with age; it is salt sensitive at the seedling stage, becomes moderately salt tolerant at the vegetative stage, and highly sensitive at reproductive stage (Lutts et al., 1995; Zeng et al., 2002). Additionally, the salt tolerance is genotype-dependent; i.e., the *Oryza sativa* Indica cultivar Nona Bokra is highly salt tolerant, the *O. sativa* Japonica cultivar Nipponbare is moderately salt tolerant, while the *O. sativa* Japonica cultivar Koshihikari is highly salt sensitive (Kurotani et al., 2015). Salt stress often causes photosynthesis decrease, plant growth inhibition, biomass loss, and partial sterility, all of which lead to yield reduction (Khatun and Flowers, 1995; Pardo, 2010; Munns, 2011; Todaka et al., 2012).

Salt tolerance is a polygenic trait controlled by several quantitative loci (QTL) (Ismail and Horie, 2017). From a mapping population derived from a *indica/indica* cross, 11 QTL for Na^+^ uptake, K^+^ uptake, and Na^+^/K^+^ selectivity in rice were mapped to regions on chromosomes (Chr.) 1, 4, 6, and 9, and the QTL for Na^+^ and K^+^ uptake were on different linkage groups (Koyama et al., 2001). In a population derived from a cross between Nona Bokra and Koshihikari, 11 QTL for salt tolerance were identified on Chr. 1, 4, 6, 7, and 9, of which two major QTL, qSNC-7 for shoot Na^+^ concentration and qSKC-1 for shoot K^+^ concentration, explained more than 40% of the total phenotypic variance (Lin et al., 2004). The major QTL Saltol, for shoot K^+^/Na^+^ homeostasis in the salt tolerant cultivar Pokkali on Chr. 1 (10.7–12.2 Mb), explained 43% of the variation for seedling shoot Na^+^/K^+^ ratio in a RIL population between indica varieties IR29 and Pokkali (Bonilla et al., 2002; Mohammadi-Nejad et al., 2008; Thomson et al., 2010). And 13 QTL for salt tolerance at seedling stage in the salt tolerantcultivar Changbai10 on Chr. 1, 5, 6, and 7 were identified by linkage mapping, of which 6 QTL were validated by association analysis of 341 japonica rice accessions (Zheng et al., 2015). Although numerous QTL for salt tolerance in rice have been identified, only SKC1, encoding an HKT-type transporter, was cloned by map-based cloning in the qSKC-1 region of Chr. 1 (Lin et al., 2004; Ren et al., 2005). Therefore, further research is needed to characterize genes for salt tolerance in rice.

Wild rice germplasm is considered a valuable source of genes for tolerance to biotic and abiotic stresses, which can be potentially used in rice breeding (Zhang and Xie, 2014; Arzani and Ashraf, 2016; Brozynska et al., 2016; Mishra et al., 2016; Menguer et al., 2017). A previous study identified 13 QTL associated with salt tolerance in the wild rice species *O. rufipogon* Griff. (Tian et al., 2011). Introgression of *PcINO1* gene that encodes L-myoinositol 1-phosphate synthase in the wild rice species *O. coarctata* was introgressed into *O. sativa* Indica cultivar and improved its salt tolerance (Das-Chatterjee et al., 2006). *OsHKT1* (high-affinity potassium transporter), *OsHKT7*, as well as numerous transcription factor genes, including zinc finger proteins (ZFPs), NAC (NAM, ATAF, and CUC), MYB, and AP2/ERF (APETALA2/ethylene response factor) are differentially expressed in Dongxiang wild rice under salt stress conditions (Zhou et al., 2016), suggesting that multiple genes are responsible for salt tolerance in wild rice.

From 1970s wild rice genes have been introgressed into cultivated rice, and most of these studies focused on the improvement resistance/tolerance to biotic stresses (Brar and Khush, 1997; Zamir, 2001; Hajjar and Hodgkin, 2007; Dempewolf et al., 2017). Therefore, information regarding the improvement of salt tolerance in cultivated rice using wild rice genes is limited.

We previously identified several genes regulating plant abiotic stress tolerance in Arabidopsis, tobacco and rice (Quan et al., 2007, 2010, 2017; Wan et al., 2011; Zhang et al., 2011). In this study, we isolated a salt tolerant introgression line Dongxiang/Ningjing 15 (DJ15) from the population of the salt-tolerant wild rice line Dongxiang (*O. rufipogon* Griff.) (Song et al., 2005; Tian et al., 2011; Zhou et al., 2016) hybridized to a cultivated rice variety (*O. sativa* ssp. japonica) Ningjing16 (NJ16). Our objectives were to (1) investigate the distribution pattern of genomic fragments introgressed into DJ15, (2) map QTL for salt tolerance, and (3) utilize newly identified QTL in rice breeding for improving salt tolerance.

## Materials and methods

### Plant materials

**Figure 1:**
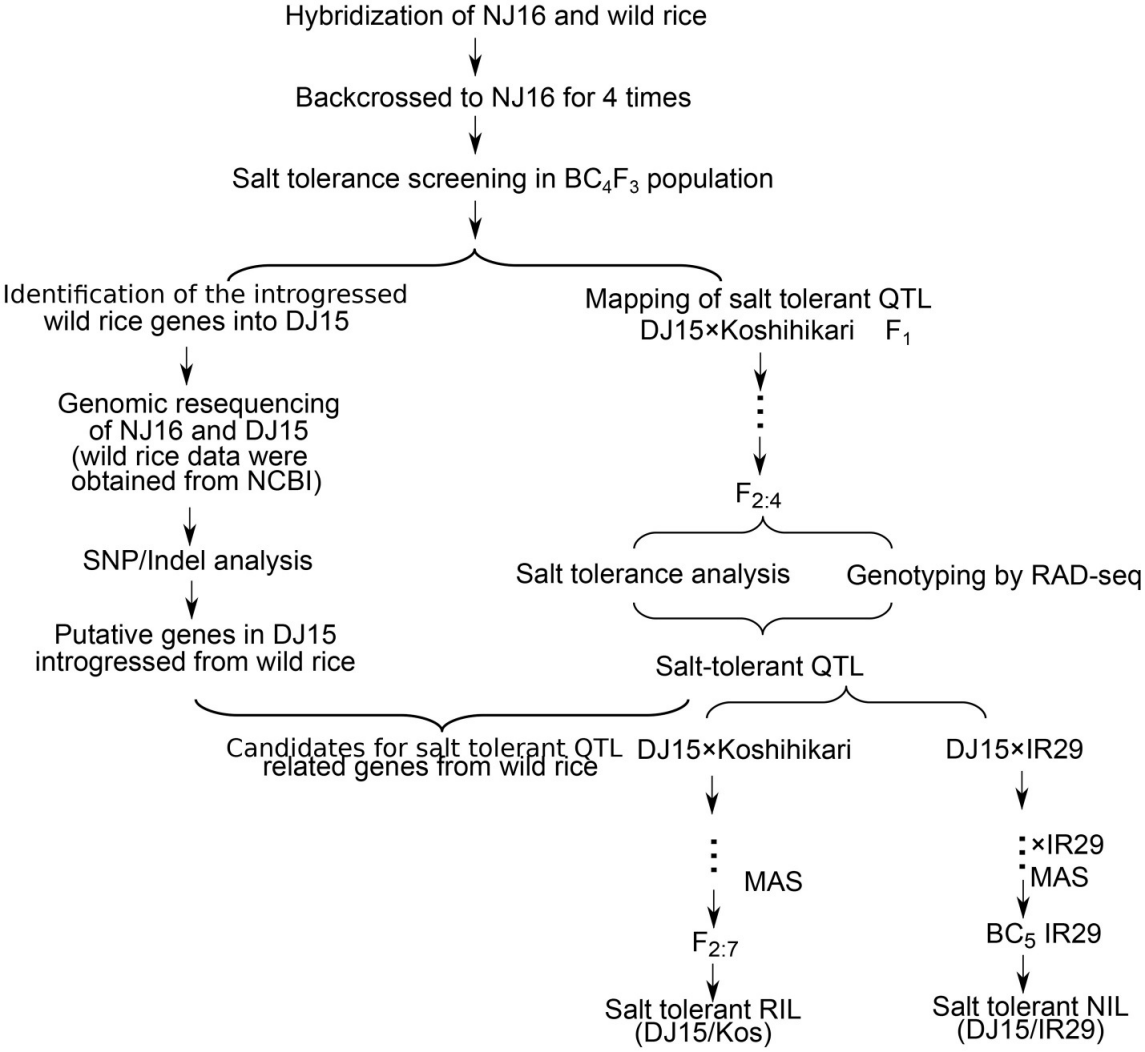
Flowchart that describes the study. Abbreviations: BC, backcross; Kos, Koshihikari; MAS, marker-assisted selection; RAD-seq, restriction-site-associated DNA sequencing.

Dongxiang wild rice, which was the northernmost population of common wild rice naturally grown in Jiangxi Province, China (N28.14) (Gao et al., 2000; Liu et al., 2017), was crossed to a cultivated japonica rice Ningjing16 (NJ16) four times (Figure 1). The salt tolerance of introgression lines was evaluated against NJ16 and a reference cultivar 96D10, which was widely planted in North China. Based on the method (Figure 1), the salt tolerant introgression line Dongxiang/Ningjing 15 (DJ15) was selected for further study.

### Determination of rice salt tolerance

Two hundred rice seeds of each sample were exposed to 45°C for 3 days, then soaked in water containing 0, 100, and 150 mM NaCl at 25°C overnight, and placed at 35°C for 3 days. And the germination rate was calculated.

Rice seeds were treated at 45°C for 3 days, soaked in water at 25°C overnight, and placed at 35°C for 3 days. Forty uniformly germinated seeds were sown on metal meshes floating on Yoshida solution (Yoshida et al., 1976) and grown at 26°C. Seedlings at 10-days stage were transferred to Yoshida solution containing 0, 100, and 150 mM NaCl for comparison among DJ15, NJ16 and 96D10, and 120 mM NaCl for F_2:4_ population. The number of wilted/dead leaves was counted 7 days later (Gao and Lin, 2013).

One-month rice seedlings were transferred to saline field [electrical conductivity of saturated soil extract (ECe) = 5.7 dS m^−1^, pH 8.45], and 1-month later the survival rates were checked. When seeds matured, agronomic parameters, including tillering number, plant height and panicle number, were determined, and the yield was calculated after harvest. ECe was determined every week, and controlled to a range of 5.7–5.9 dS m^−1^ by the irrigation of fresh water or saline water as required.

### Analysis of the distribution of single nucleotide polymorphism (SNP)/ insertion or deletion (Indel) variants

Genomic resequencing of DJ15 and NJ16 was conducted by a high-throughput sequencing platform (Hiseq2500, Illumina, Inc.; San Diego, CA, U.S.), and the resequencing data were deposited in the National Center for Biotechnology Information Sequence Read Archive (NCBI SRA) under the accession numbers SRR6423773 and SRR6423774. The resequencing data of Dongxiang wild rice was obtained from NCBI SRA accession SRX158100. The pair-end reads were mapped to Nipponbare rice reference genome (MSU Rice Genome Annotation Project Release 7) (Kawahara et al., 2013) by BWA (Li and Durbin, 2009). Then SNP/Indel variants were called using GATK (McKenna et al., 2010; DePristo et al., 2011). Finally, SNP/Indel density was calculated from the variation data by VCFtools (Danecek et al., 2011).

To verify the Indel variants derived from resequencing analysis, we designed specific PCR primers for randomly selected Indel variants with 3bp or more difference, spreading over the 12 chromosomes (Table S1). PCR amplification was conducted using genomic DNA isolated from NJ16, DJ15 and Dongxiang wild rice as templates. And the PCR products were separated by electrophoresis in 4% agarose gel.

### Generation of QTL mapping population

DJ15 was crossed with the salt sensitive and late maturing Japonica cultivar Koshihikari. F_3_ and F_4_ were obtained by harvesting separately from responding F_2_ parents, which were named as F_2:3_ and F_2:4_, respectively.

DJ15 was backcrossed to a salt sensitive indica rice variety IR29 (recurrent parent) five times, along with the confirmation of QTL in the offspring by marker assisted screening at each generation.

### Population genotyping by restriction-site-associated DNA sequencing (RAD-seq)

Genomic DNA was extracted from 103 F_2:4_ lines and the two parental lines using the cetyl trimethylammonium bromide (CTAB) method (Murray and Thompson, 1980). Sequencing libraries were constructed from the genomic DNA using the method modified from previous description (Sun et al., 2013). In brief, genomic DNA was incubated at 37°C with *Mse*I, T4 DNA ligase, ATP and *Mse*I adapter. After heat-inactivation of the restriction/ligation reactions at 65°C, the samples were digested with *Rsa*I and *Hpy*166II, then the subsequent steps for library construction were carried out as detailed in previous description (Sun et al., 2013). Then, pair-end sequencing was performed using a high-throughput sequencing platform (Hiseq2500, Illumina, Inc.; San Diego, CA, U.S.). Finally, specific-locus amplified fragments (SLAFs) were processed to obtain the SLAF genotype of each sample (Sun et al., 2013).

### QTL analysis

A total of 5,472 polymorphic SLAFs was obtained from the RAD-seq data of DJ15 and Koshihikari. Using HighMap method as described by Liu et al. (2014), a high density linkage map was constructed from the SLAF genotyping data of 103 F_2:4_ lines. Next, QTL were identified by the composite interval mapping method using Windows QTL Cartographer (Silva et al., 2012). The LOD threshold of QTL significance was determined by a permutation test (1,000 replications) at *P* < 0.05.

### Identification of candidate genes for salt tolerance in QTL

The genomic resequencing data of Koshihikari was obtained from NCBI (DRX002963). After aligning of the pair-end reads to rice reference genome (MSU Rice Genome Annotation Project Release 7) (Kawahara et al., 2013) using BWA (Li and Durbin, 2009), SNP/Indel variants were called using GATK (McKenna et al., 2010; DePristo et al., 2011). The different SNP/Indel variants of DJ15, Koshihikari, NJ16, and Dongxiang were processed with GATK (McKenna et al., 2010; DePristo et al., 2011), and annotated with SnpEff (Cingolani et al., 2012). Candidate genes for salt tolerance in the QTL regions were obtained after filtering out transposons and pseudogenes.

## Results

### Evaluation of DJ15 for salt tolerance

**Figure 2:**
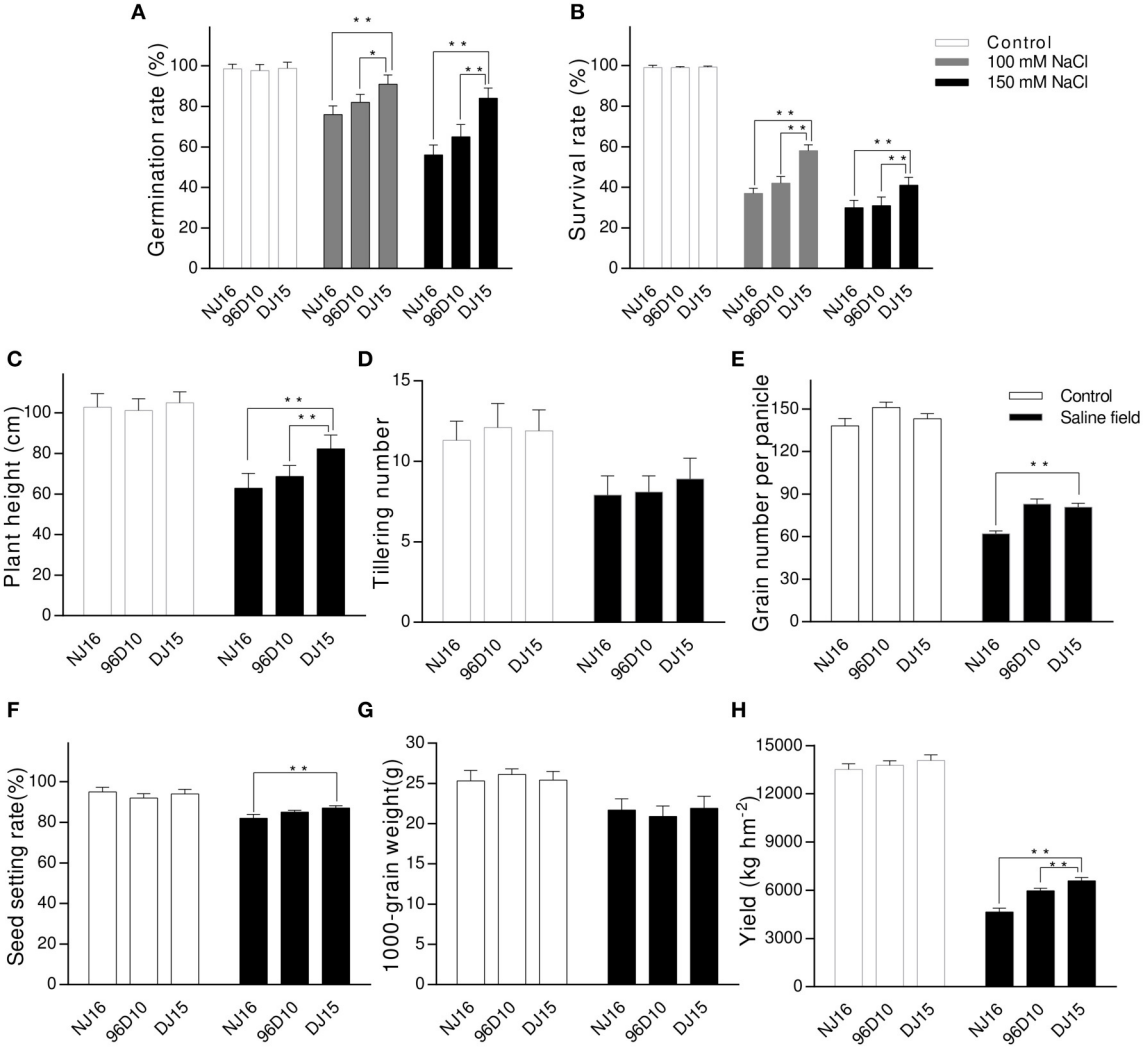
Salt tolerance evaluation of introgression line DJ15. **(A)** Germination rate of rice seeds after treatment at 0, 100, and 150 mM NaCl for 3 days at 35°C. **(B)** Survival rate of 10-day-old seedlings after treatment at 0, 100, and 150 mM for 10 days and subsequent recovery for 7 days. **(C–H)** Field performance of rice under non-stress field conditions (ECe = 1.3 dS m^−1^, pH 7.01) and under salt-stress field conditions (ECe = 5.7 dS m^−1^, pH 8.45). ^*^Indicates significant difference at *P* < 0.05 in Tukey's multiple comparisons test after ANOVA; ^**^Indicates significant difference at *P* < 0.01 in Tukey's multiple comparisons test after ANOVA.

The salt-tolerant introgression line DJ15 was selected by lab screening and field evaluating the populations of Dongxiang/Ningjing16 (Ningjing16 as the recurrent parent) at backcross2 (BC_2_) to BC_4_ generations (Figure 1). Under non-stress conditions, the germination rate of DJ15 was not significantly different from that of the parental variety NJ16 and the control variety 96D10 (Figure 2A). However, at the 150 mM NaCl treatment, the germination rate of DJ15 was reduced by 16%, of NJ16 by 44% and of 96D10 by 35%, suggesting that DJ15 was more salt tolerant than NJ16 and 96D10 at the germination stage.

At the 100 and 150 mM NaCl treatment, the percentage of wilted/dead leaves was 59 and 42% for DJ15, 37 and 30% for NJ16, and 41 and 31% for 96D10, respectively, indicating that DJ15 was also more salt tolerant than NJ16 and 96D10 at the seedling stage (Figure 2B).

Under non-stress field conditions (ECe = 1.3 dS m^−1^, pH 7.01), the yield of DJ15 was slightly higher than that of NJ16 and 96D10; however, plant height, 1,000-grain weight, grain number per panicle, and effective tillering per plant were not significantly different among DJ15, NJ16 and 96D10. Under salt-stress field conditions (ECe = 5.7 dS m^−1^, pH 8.45), the yield of DJ15 was markedly higher than that of NJ16 and 96D10, possibly due to its higher plant height and effective tillering number, grain number and seed setting rate (Figures 2C–H).

Therefore, these results suggested that DJ15 was more salt tolerant than NJ16 and 96D10 at the germination, seedling and mature stages (Figure 2).

### Identification of introgressed genomic fragments in DJ15

By comparing SNP/Indel variants of DJ15, NJ16 and Dongxiang wild rice, we found 642,349 SNP/Indel variants between DJ15 and NJ16 across the 12 chromosomes, among which 265,862 variants might be obtained from Dongxiang wild rice (Table 1). More than 97% of 617,227 SNP/Indel variant effects were in intergenic, intron, downstream and upstream of the coding regions, whereas only 2.3% in exons (Table 2).

**Table 1:** Number of SNP/Indel variants in the *O. rufipogon* line Dongxiang, the *O. sativa Japonica* variety Ningjing16 (NJ16), and the salt-tolerant introgression line DJ15.

**Chromosome**	**DJ15 vs. NJ16**	**(DJ15 = Dongxiang) vs. NJ16**
1	29,164	11,550
2	41,189	12,497
3	14,797	6,090
4	22,665	13,017
5	34,779	9,396
6	156,245	85,301
7	62,336	21,950
8	25,905	11,802
9	18,535	9,817
10	64,841	19,714
11	158,873	59,294
12	13,020	5,434
Total	642,349	265,862

**Table 2:** Number of SNP/Indel effects by genomic region.

**Type (alphabetical order)**	**Count**	**Percent(%)**
Downstream	138,852	22.496
Exon	14,534	2.355
Intergenic	217,168	35.184
Intron	33,819	5.479
Splice_site_acceptor	39	0.006
Splice_site_donor	32	0.005
Splice_site_region	805	0.13
Transcript	53,643	8.691
Upstream	145,527	23.578
Utr_3_prime	7,861	1.274
Utr_5_prime	4,947	0.801
Total	617,227	100%

**Figure 3:**
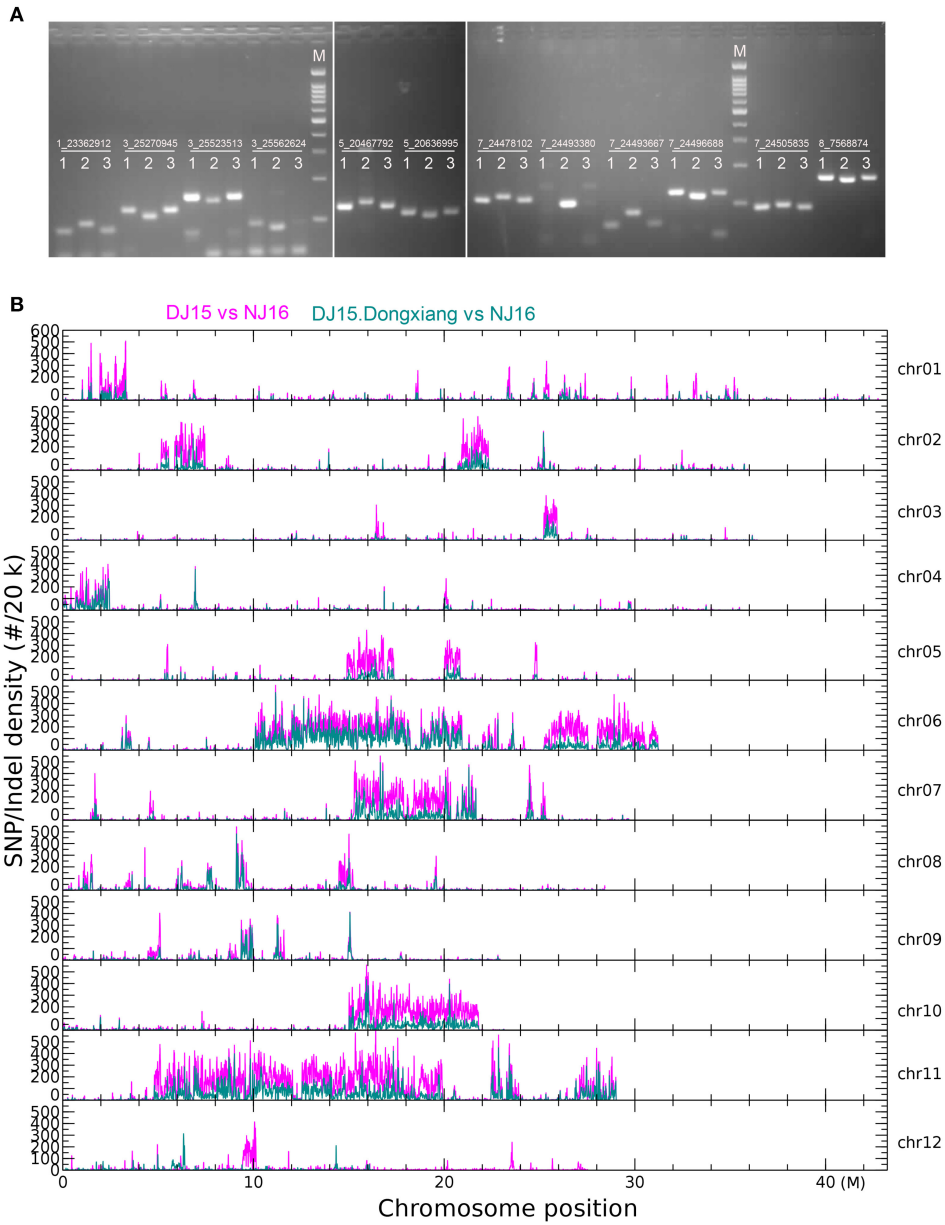
Distribution of single nucleotide polymorphism (SNP)/insertion or deletion (Indel) variants in rice genome. **(A)** PCR verification of Indels with 3 bp or more difference among the *O. rufipogon* line Dongxiang (1), the *O. sativa Japonica* variety Ningjing16 (2), and the salt-tolerant introgression line DJ15 (3). M. 100 bp ladder. **(B)** Density of SNP/Indel variants in rice genome. “DJ15 vs. NJ16” indicates SNP/Indel variants between DJ15 and NJ16; “DJ15.Dongxiang vs. NJ16” indicates SNP/Indel variants identical between DJ15 and Dongxiang, but different from NJ16.

PCR amplification using specific primers for 52 randomly selected Indel variants showed that different fragments were amplified from the genomic DNA of DJ15, NJ16, and Dongxiang (Figure 3A, Figure S1; Table S1). This result indicated that the identified variants from high-throughput sequencing were accurate.

The rate of variants between NJ16 and Dongxiang wild rice was one variant for every 194 bases, which was markedly higher than that between NJ16 and DJ15 (one variant for every 581 bases). Therefore, the genomic regions introgressed from Dongxiang wild rice into DJ15 were probably enriched with SNP/Indel variants. The regions of high density in SNP/Indel variants between DJ15 and NJ16 (receipt line) distributed unequally over the 12 rice chromosomes, within which four large regions of high density were located in Chr. 6, 7, 10, and 11 (Figure 3B). The distribution pattern of SNPs/Indels was identical between DJ15 and Dongxiang wild rice, different between NJ16 and Dongxiang, and similar between DJ15 and NJ16. These results indicated that the regions of high density in SNP/Indel variants identified in DJ15 might be large DNA fragments introgressed from Dongxiang wild rice.

### QTL mapping

**Figure 4:**
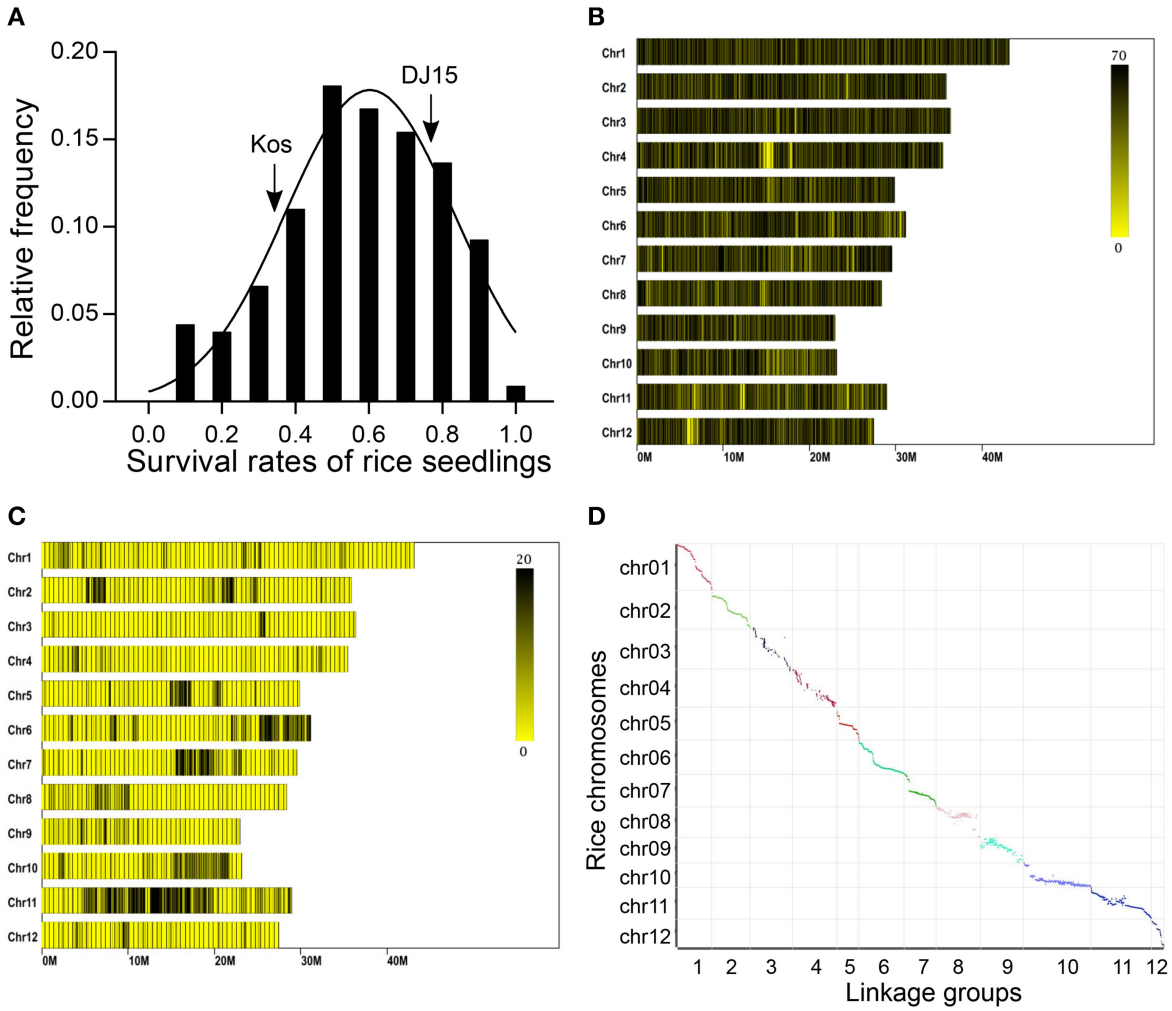
Salt tolerance screening and genotyping of DJ15 × Koshihikari F_2:4_ rice population. **(A)** Survival rate of 2-week rice seedlings after treatment at 120 mM NaCl for 10 d. Arrows indicate the survival rate of DJ15 and Koshihikari (Kos), respectively. **(B)** Distribution of specific-locus amplified fragments (SLAFs) in the rice reference genome. **(C)** Distribution of polymorphic SLAFs between DJ15 and Koshihikari in rice reference genome. Color bars indicate the SLAF number in the rice genome within 1 M window. **(D)** Comparison of the linkage map constructed from DJ15 × Koshihikari F_2:4_ population with the rice reference physical map.

QTL for salt tolerance were mapped in an F_2:4_ population (*n* = 103) derived from a cross between DJ15 and a salt sensitive japonica rice variety Koshihikari. Lab and field screening for salt tolerance showed that the survival rate of DJ15 seedlings was 75%, whereas that of Koshihikari was 37%. Curve fitting by Gaussian method showed that the distribution of salt tolerance in F_2:4_ lines was close to normal with a slight skewness to the salt sensitivity side, since 61% of the lines congregated between DJ15 and Koshihikari, 15% of the lines were more salt sensitive than Koshihikari, and 24% of the lines were more salt tolerant than DJ15 (Figure 4A).

We used restriction-site-associated DNA sequencing to determine the genotype of DJ15, Koshihikari and the F_2:4_ lines. DJ15 and Koshihikari were sequenced at an average depth of 23.98 ×, whereas F_2:4_ lines were sequenced at an average depth of 4.18 ×. We found a total of 136,724 specific-locus amplified fragments (SLAFs), of which 5.49% were polymorphic SLAFs (Tables S2, S3).

Mapping SLAFs to the rice reference genome (MSU Rice Genome Annotation Project Release 7, http://rice.plantbiology.msu.edu/) showed that they were nearly equally distributed over the 12 rice chromosomes. However, polymorphic SLAFs were unequally distributed over the chromosomes and condensed to six regions of high density on Chr. 2, 5, 6, 7, 10, and 11 (Figures 4B,C).

After filtering SLAFs with sequencing depth under 10 × or heterozygous in parents, we obtained 5,479 polymorphic SLAFs. Then using HighMap software (Liu et al., 2014), we constructed a high density linkage map from SLAF-seq genotyping data of DJ15 × Koshihikari F_2:4_ lines. After filtering makers with MLOD <5, we arranged 5,472 of 5,479 SLAF markers to 12 linkage groups, with an average distance of 0.34 cM between two adjacent markers. The linkage map was consistent with the rice reference physical map as revealed by mapping SLAFs to the rice reference genome and finding a Spearman's correlation coefficient of approximately one (Figure 4D). Thus, the marker positions on the linkage map were valid and the genotyping results were accurate.

Using composite interval mapping, we found 9 QTL for salt tolerance spreading over 7 chromosomes with an LOD cutoff > 3.0 (Table 3). The positive additive effects of 7 QTL were derived from DJ15 alleles, and the total phenotypic variance explained by each QTL was 8.81–22.10%. Of these, qST6 accounted for 19.38% of the total phenotypic variance, and had an additive effect of 0.12 for increasing survival rate derived from DJ15 allele.

**Table 3:** QTL for salt tolerance identified in DJ15 × Koshihikari F_2:4_ segregating population.

**QTL**	**Chromosome**	**Chromosome position (M)**	**Peak LOD**	**Additive effect^a^**	**R^2^(%)^b^**
qST1.1	1	40.3	4.90	0.017	22.10
qST1.2	1	10.6	3.30	0.038	10.00
qST3	3	6.2	3.61	0.022	14.27
qST4.1	4	29.6	3.65	0.15	9.79
qST4.2	4	23.4	3.55	−0.096	8.81
qST5	5	16.5	3.98	−0.11	12.67
qST6	6	25.6	4.12	0.12	19.38
qST8	8	21.7	4.33	0.019	15.80
qST10	10	17.2	3.68	0.13	11.35

a *Additive effect on the DJ15 allele*.

b *Percentage of total phenotypic variance explained by the QTL*.

### Candidate genes for salt tolerance in QTL

To identify candidate genes for salt tolerance in the QTL regions, we analyzed the sequence variants of NJ16, DJ15, Koshihikari and Dongxiang wild rice in the 9 QTL regions (Table S4). Among the candidate genes, we found *Os01g0307500* (*OsSKC1*) located in the qST1.2 region and *Os01g0932500* (*OsHAK6*) located in the qST1.1 region. Other QTL candidates included several transcription factors (MYB, zinc finger and AP2/ERF) and protein kinases, which might stimulate plant salt stress tolerance by transcriptional regulation and kinase pathways (Golldack et al., 2014). These results indicated that the identified QTL might enhance rice salt tolerance via ionic homeostasis maintenance, gene transcription and kinase signaling pathways.

### Application of QTL for improvement of salt tolerance in cultivated rice

As DJ15 is a salt tolerant and early maturing introgression line which is only suitable for Ningxia but not suitable for planting in other areas of Northeast China, we investigated if it would be possible to identify a highly salt-tolerant and late maturing line by hybridization of DJ15 to a salt sensitive but late maturing rice variety Koshihikari. By marker-assisted screening, we identified 55 lines carrying qST1.2^DJ15^ and qST6^DJ15^ from 980 DJ15/Koshihikari recombinant inbred lines (RILs). Under salt-stress field conditions (ECe = 4.0 dS m^−1^), the heading dates of 6 (YD7, 8, 22, 23, 28, 44) of 55 lines were about 4–11 days later than that of DJ15, and their yields were 8–23% more than that of DJ15 or 36–59% more than that of Koshihikari (Table 4). Therefore, the 6 salt tolerant, high yielding and late maturing lines might be used for developing new rice varieties.

**Table 4:** Agronomic traits of DJ15/Koshihikari recombinant inbred lines (RILs) carrying qST6^DJ15^ under salt-stress field conditions (ECe = 4.0 dS m^−1^, pH 8.5).

**RIL**	**Tillering No**.	**Panicle No**.	**Plant height(cm)**	**Heading date**	**Yield (kg hm^−2^)**
YD7	10.2	9.2	94.7	Aug 1	11,743
YD8	9.2	7.8	93.6	Aug 2	11,411
YD22	13.0	11.0	97.2	Aug 5	10,085
YD23	12.0	11.0	94.0	Aug 4	10,275
YD28	14.4	12.2	98.4	Aug 8	10,511
YD44	10.0	9.4	97.3	Aug 2	10,606
DJ15	13.8	11.6	88.7	Jul 28	9,521
Koshihikari	16.4	14.4	94.3	Aug 9	7,386

**Figure 5:**
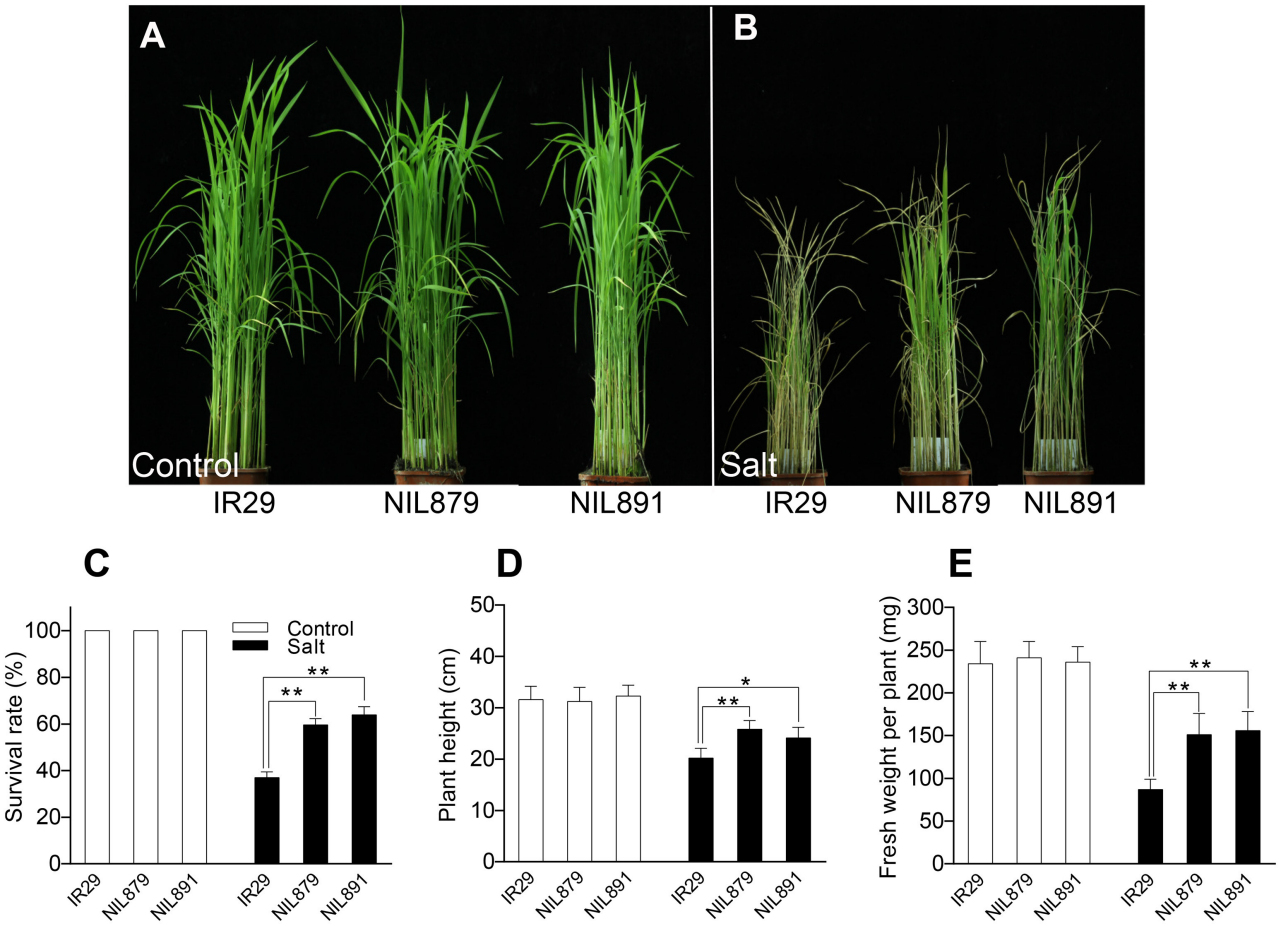
Salt tolerance of qST6^DJ15^ near isogenic lines. **(A–E)** Survival rate, plant height, and fresh weight of 2-week-old IR29 and qST6^DJ15^ NILs (NIL879 and NIL891) seedlings after treatment at 80 mM NaCl for 10 days and subsequent recovery for 5 days. ^*^Indicates significant difference at *P* < 0.05 in Tukey's multiple comparisons test after ANOVA; ^**^Indicates significantly difference at *P* < 0.01 in Tukey's multiple comparisons test after ANOVA.

qST1.2 and qST6 were two QTL that showed the highest effect for salt tolerance in DJ15, and SKC1 might be one major effect gene for qST1.2 (Ren et al., 2005). Among the 9 newly identified QTL for salt tolerance, qST6 was a novel QTL in a region of high SNP/Indel variant density; thus, we backcrossed DJ15 to IR29 5 times and screened the offspring by qST6 linked markers to select IR29 near-isogenic line (NIL)^DJ15^. Under non-stress conditions, no significant differences were found in the seedling growth of IR29 and IR29 NIL^DJ15^ (Figure 5). Lab screening under salt stress conditions (80 mM NaCl) for 10 d, IR29 NIL^DJ15^ seedlings demonstrated higher survival rate, plant height, and biomass compared with IR29, indicating that IR29 NIL^DJ15^ was more tolerant to salt stress (Figure 5). These results suggested that qST1.2 and qST6 could be utilized in rice breeding for improving salt tolerance.

## Discussion

*O. sativa* and *O. glaberrima* are the only two domesticated species within the genus *Oryza*, and thus the genetic diversity of cultivated rice is limited. *Oryza* contains more than 20 wild species, which are considered a significant source of genetic diversity (Atwell et al., 2014) since they compose a valuable pool of genes for disease or abiotic stress resistance/tolerance (Brar and Khush, 1997; Zamir, 2001; Hajjar and Hodgkin, 2007; Maxted et al., 2012; Mishra et al., 2016; Dempewolf et al., 2017; Wang et al., 2017). Pest/disease resistance is the most well studied trait that can be improved by introgression of wild rice genes (Hajjar and Hodgkin, 2007). In addition, the genome of wild rice contains tolerant alleles of abiotic stress genes. For example, submerge tolerant *O. rufipogon* and *O. nivara* accessions carry the *SUB1A-1* allele for submergence tolerance (Niroula et al., 2012). An accession of wild rice (*O. rufipogon*) from Yunnan Province of China contains 13 QTL for salt tolerance QTL (Tian et al., 2011). Dongxiang wild rice changes the expression of more than 6,800 genes related to salt stress, including numerous transcription factor genes, such as ZFP, NAC, MYB, and ERF, indicating that these transcription factors might regulate multiple regulatory pathways under salt stress conditions (Zhou et al., 2016). However, information regarding the improvement of salt tolerance in cultivated rice using wild genes is limited. In this study, we demonstrate that wild rice genes could enhance the salt tolerance of cultivate rice.

NJ16 is a commercial variety widely cultivated in the Yellow-river irrigating region of Ningxia Province in China. However, the yield is negatively affected by salt stress due to the widespread saline soil in this region. In the present study, we introgressed wild rice fragments into a rice cultivar, and obtained an introgression line with improved salt tolerance after several rounds of selection. These results demonstrated that wild rice genes could be used to improve salt tolerance in cultivated rice.

AtHKT1 promotes Na^+^ unloading from the xylem and increases salt tolerance in Arabidopsis (Davenport et al., 2007; Møller et al., 2009). OsSKC1 (Ren et al., 2005) is a HKT-like protein, namely HKT8/HKT1;5 (Garciadeblás et al., 2003; Hauser and Horie, 2010). OsSKC1 is not primarily a K^+^ transporter but is a Na^+^ selective transporter, which regulates Na^+^/K^+^ levels in shoots by recirculating Na^+^ from shoots to roots, thus affecting the shoot K^+^ content indirectly (Ren et al., 2005). Furthermore, Immuno-staining and^22^Na^+^ tracer experiments suggested that OsHKT1;5 protein contributes to Na^+^ exclusion in the phloem, in addition to xylem Na^+^ unloading, to prevent Na^+^ transfer to young leaf blades (Kobayashi et al., 2017). K^+^ transporter OsHAK1 mediates K^+^ uptake in roots and K^+^ translocation to shoots, which could enhance salt tolerance (Bañuelos et al., 2002; Chen et al., 2015). Similarly, OsHAK5 enables K^+^ uptake by roots under external K^+^ nutrient limitation and saline conditions (Yang et al., 2014). Therefore, OsHAK6 in qST1.1 might mediate salt tolerance as the OsHAKs mentioned above.

Salt tolerance is a complex trait controlled by many genetic loci. For example, eleven QTL related to salt tolerance are located on 5 chromosomes from the F_3_ population of Nona Bokra and Koshihikari (Ren et al., 2005). And 13 of 15 QTL on six chromosomes from wild rice improve salt tolerance in Teqing background (Tian et al., 2011). Compared with salt tolerance QTL identified from wild rice *O. rufipogon* (Tian et al., 2011), in this study the position of qST1.2 is close to qSTS1, and the other 5 QTL for salt tolerance are novel. Up to now, only *SKC1* localized in qST1.2 region has been cloned by mapping (Ren et al., 2005). Therefore, the function of other candidate genes for salt tolerant QTL remains to be explored in future studies.

Although several QTL for salt tolerance have been identified from wild rice (Tian et al., 2011), their physiological function has not been confirmed under salt-stress field conditions. In the present study, we obtained 6 salt-tolerant DJ15/Koshihikari RILs, carrying both qST1.2^DJ15^ and qST6^DJ15^, via marker-assisted screening. These RILs showed a lower tillering and panicle number than Koshihikari and DJ15, but a higher yield, under salt-stress field conditions, which might result from the relatively higher seed setting rate of RILs. Additionally, IR29 NIL^DJ15^ was more salt tolerant than IR29 at the seedling stage and showed higher yield per plant in a preliminary study. However, more research is needed to elucidate the underlying regulating mechanism of yield in relation to qST6^DJ15^ under salt stress conditions.

As DJ15 and the salt tolerant RILs demonstrate variation in growth duration, the growth adaptability in saline field of Dongying (Shandong Province, E118.7, N37.6) and Panjin (Liaoning Province, E122.0, N41.0), other than Yinchuan (Ningxia Province, E106.2, N38.5) in North China, is under investigation. And we hope to release these salt tolerant lines to the public in the near future. Overall, our results demonstrated that wild rice genes could be successfully used in rice breeding programs to develop varieties with markedly improved salt tolerance.

## Author contributions

RH conceived the project. SL, JH, HB, XL, and YZ constructed wild rice introgression lines to isolate DJ15, and performed saline field tests. JW performed salt tolerance determination by hydroponics in F_2:4_ population. RQ performed the rest of experiments and analyzed data. HZ and ZZ analyzed the data. RQ wrote the manuscript.

### Conflict of interest statement

The authors declare that the research was conducted in the absence of any commercial or financial relationships that could be construed as a potential conflict of interest.
