# Supplementary material for: Improvement of Salt Tolerance Using Wild Rice Genes
Source: Front Plant Sci. 2018 Jan 17;8:2269. doi: 10.3389/fpls.2017.02269 (PMC5776132; doi:10.3389/fpls.2017.02269)
Supplement: Supplementary file 1 [file Image1.pdf]

Supplementary Material:

Introgression of Wild Rice Genes Improves Salt Tolerance in Cultivated Rice

Ruidang Quan, Juan Wang, Jian Hui, Haibo Bai, Xuelian Lyu, Yongxing Zhu, Haiwen Zhang, Zhijin Zhang, Shuhua Li* and Rongfeng Huang*

Correspondence: Rongfeng Huang, email: rfhuang@caas.cn, and Shuhua Li, email: shuhua.l@163.com

SUPPLEMENTARY FIGURES AND TABLES

Figure S1 Snapshots of SNP/Indel variants in representative genomic regions of rice.

Table S1 Specific primers designed for confirming Indel variants.

Table S2 Statistics of specific-locus amplified fragments (SLAFs).

Table S3 Statistics of different types of specific-locus amplified fragments (SLAFs).

Table S4 Candidate genes for salt tolerance in QTL.

chr01_23 362 912

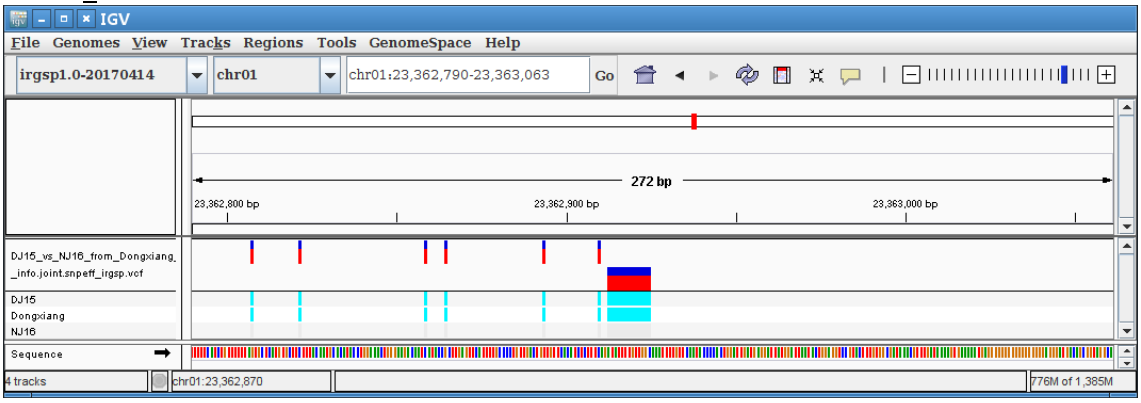

chr03_25 270 945

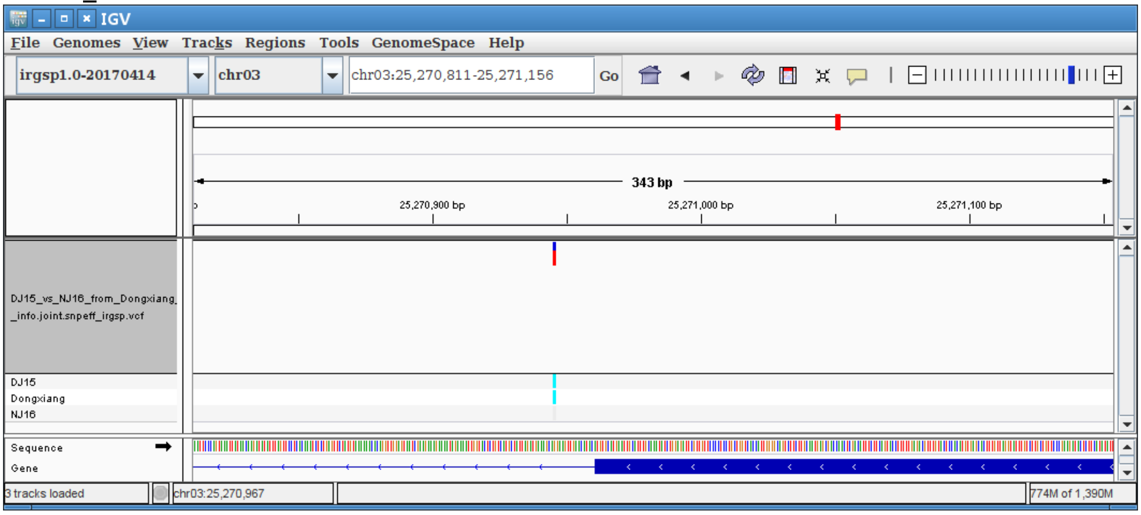

chr03_25 523 513

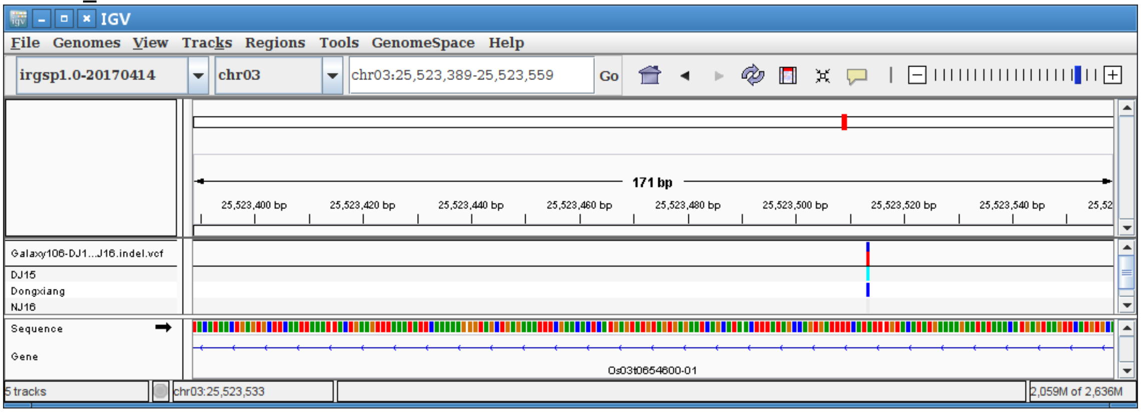

Figure S1 Snapshots of SNP/Indel variants in representative genomic regions of rice. Variant Call Format (VCF) file containing variants among DJ15, NJ16 and wild rice Dongxiang was viewed with Integrative Genomics Viewer (IGV) (http://software.broadinstitute.org/software/igv/home).

chr03_25 562 624

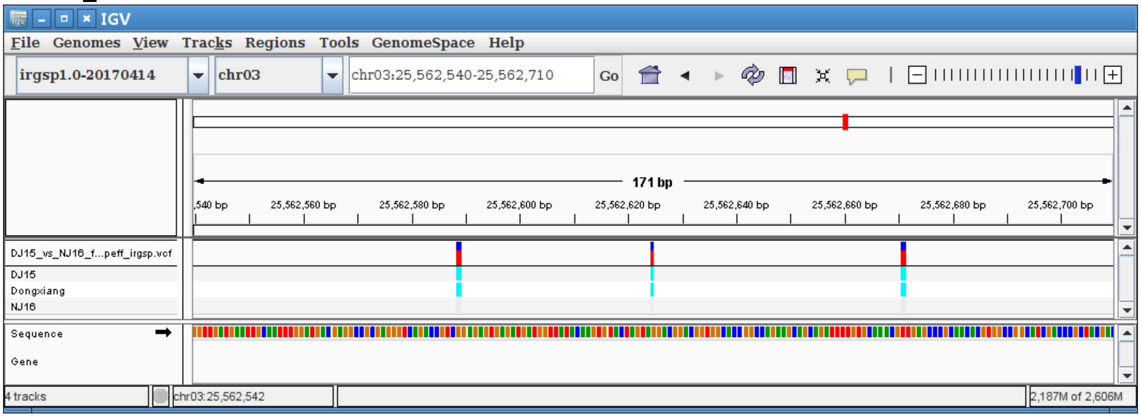

chr05_20 467 792

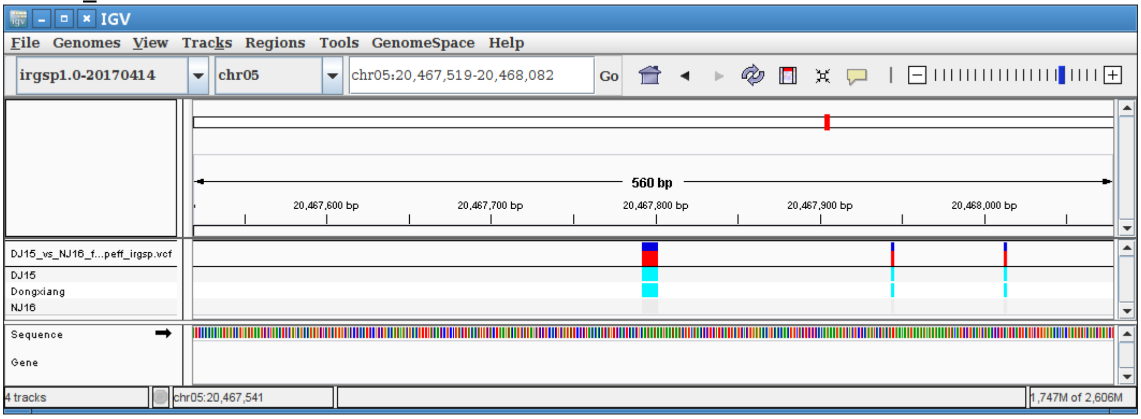

chr05_20 636 995

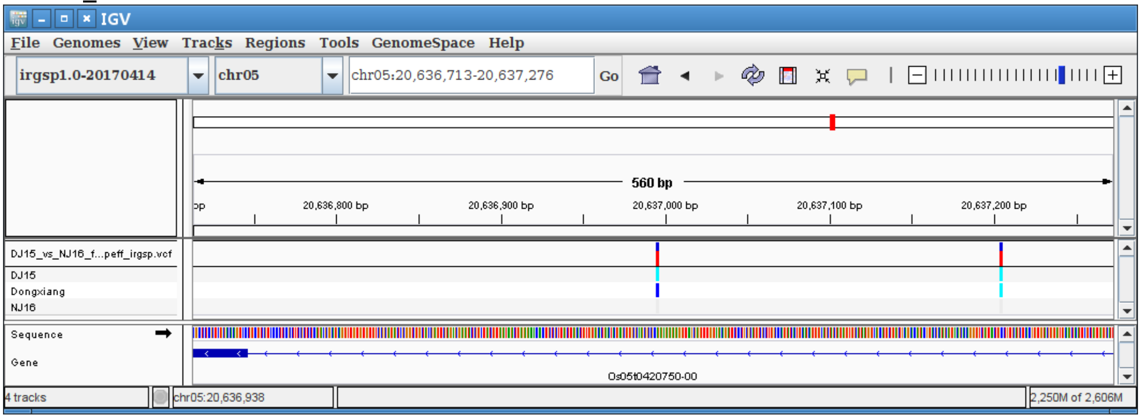

Figure S1 (continued)

chr07_24 478 102

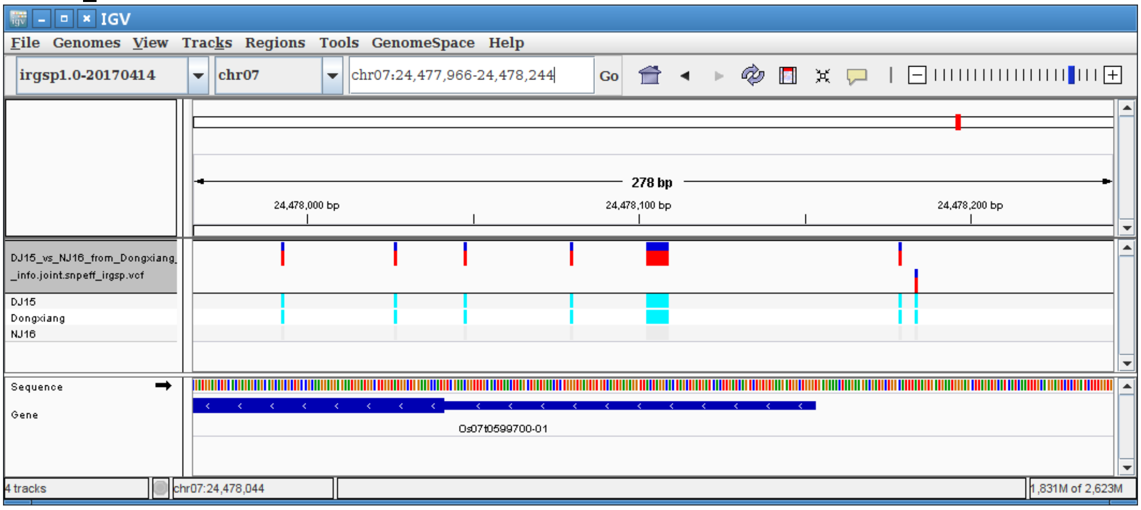

chr07_24 493 380

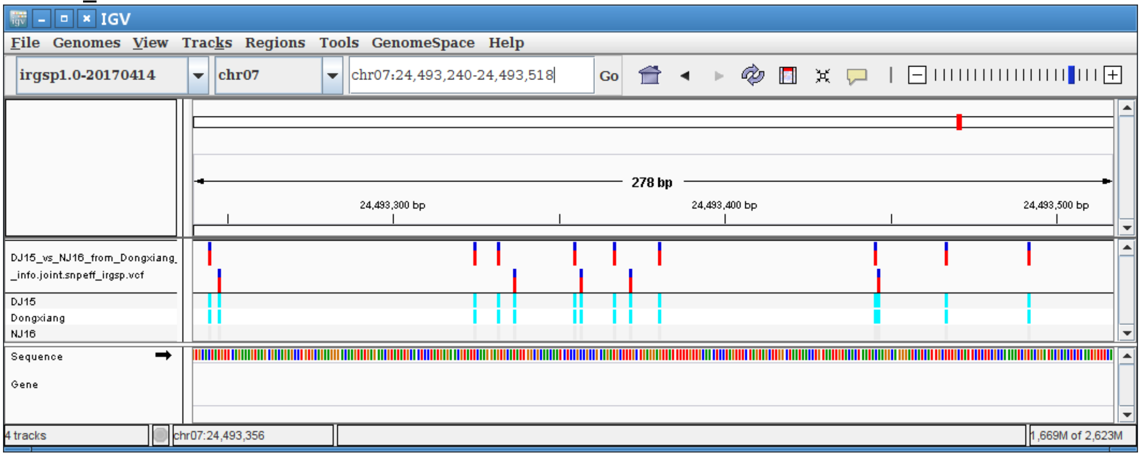

chr07_24 493 667

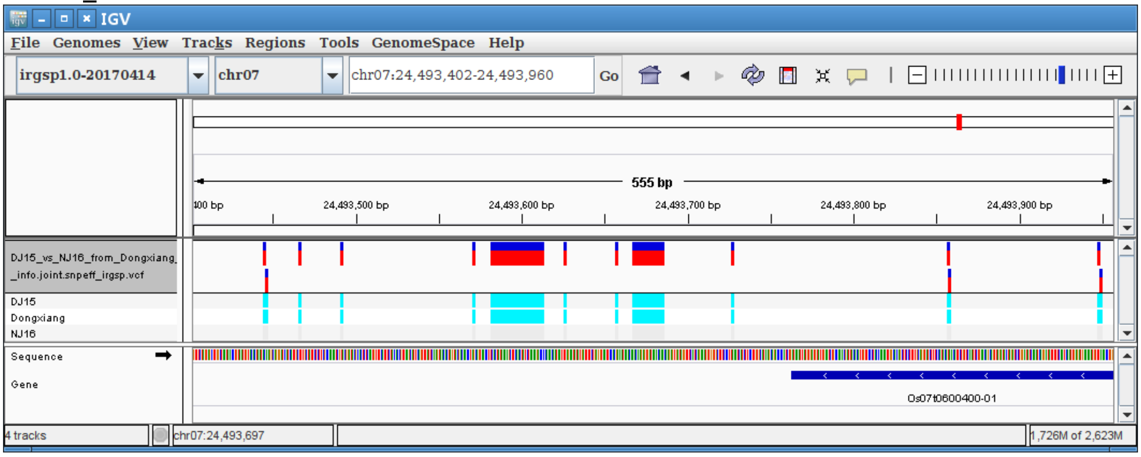

Figure S1 (continued)

chr07_24 496 688

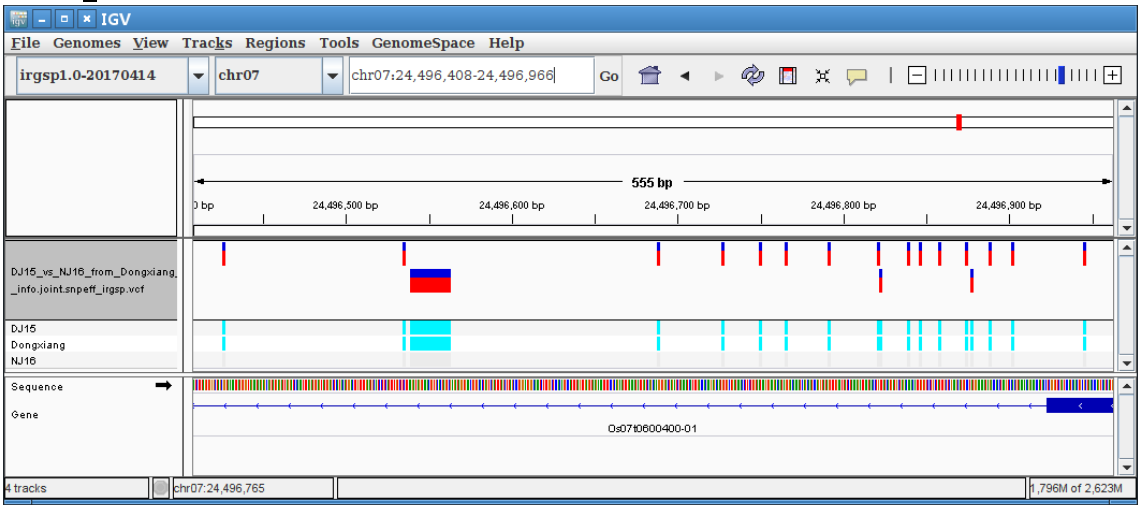

chr07_24 505 835

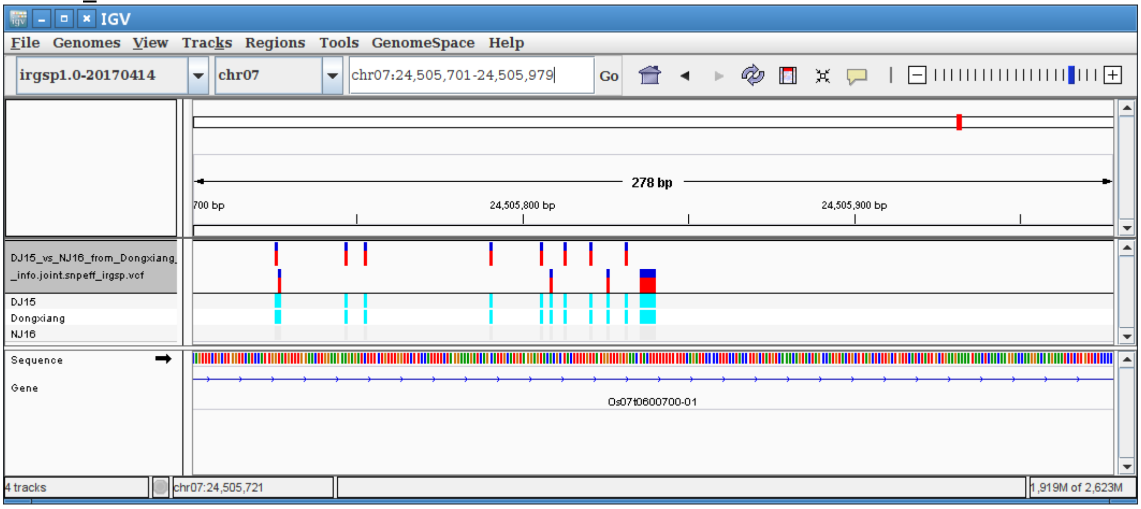

chr08_7 568 874

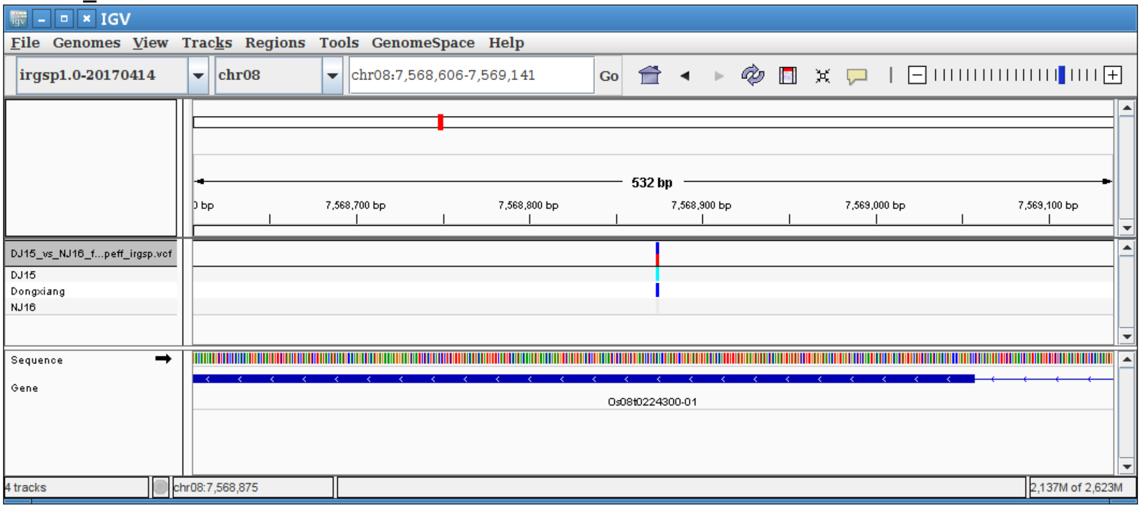

Figure S1 (continued)

Table S1 Specific primers for confirmating Indel variants.

Name	Sequence (5' to 3')
chr01_23362912F	ACCGCTTGAGTTCATTTTGC
chr01_23362912R	CATCAATCCAGTGGGGTATTG
chr03_25270945F	CACAGGTTGGAGTAATCGAAGA
chr03_25270945R	TTTGCAAATGCAATTCATGC
chr03_25523513F	GCTGAGAAATTTCGAACACTCA
chr03_25523513R	AATCACCCCATCATCATCAAA
chr03_25562624F	CCGTCGGAGAGTGAGATGAT
chr03_25562624R	TGCACAAAATCTGCTGCTTC
chr05_20129867F	TTAGTGGCCCAAATTCGGTA
chr05_20129867R	CCACCGAGAATGGCAATAGT
chr05_20467792F	TCCTTGCCACCTGCTAGTGT
chr05_20467792R	CCTCCACAGTGAACTGCAAA
chr05_20636995F	AACCAACCTTGCTCGCTTTA
chr05_20636995R	CCTCGCTTAAACCCTCTCTC
chr07_24320487F	TGCACTCCAAACCAGCAATA
chr07_24320487R	TCGGTGGTTAGATGCAATTCT
chr07_24478102F	TGTCGAACGGTTTGCACTTA
chr07_24478102R	TTTCTCACCCAGCCATCTTC
chr07_24493380F	GCAGATGCTTTGCTGAGGA
chr07_24493380R	AATGATGATCCCTGCCTTGT
chr07_24493667F	TGAGGTAATTCCACGAAATAAACA
chr07_24493667R	GGGAAAGCACCACATTTCTG
chr07_24496688F	CTTTGGCTCCACAGTCCATT
chr07_24496688R	GCAACATTGCTCTTGTGGTC
chr07_24505835F	GGAAAGATCAGCATTGCATGT
chr07_24505835R	GCAAGGTAGGAAAGGGGAAT
chr08_7568874F	CTGGGACAAAATCGCTCATT

to be continued...

Name	Sequence (5' to 3')
chr08_7568874R	GCCCTAACCAGTGCTTACCC
chr08_7619086F	AAAATGCATAGCAGCAATCG
chr08_7619086R	TCTTGTTCGGCGTTTCTTTT
chr08_7636199F	CCACTAGGATTTTGTCCACGTC
chr08_7636199R	CACAGAAGCCATGAAGGTCA
chr08_7798616F	CGACGAATCCATCACTTCTG
chr08_7798616R	TGTACACCATCATCGCATCA
chr08_9124295F	GGCAGAGAATGGCAATGAAT
chr08_9124295R	CAGGCCACCTTTCCCATATC
chr08_9129222F	AGTTCAGCCGTGAGACTTCA
chr08_9129222R	CCTTGCATAAGTCACTGTTCTCC
chr08_9129318F	TGCAGAGTTCAAACTGGAGAA
chr08_9129318R	CCCTGTACCATCAAGGCAAG
chr08_9133389F	ATTTGGGTTGAGGTGTCCAA
chr08_9133389R	GCTCTGCTTCGGTTAAATGC
chr08_9133740F	GCAATGCATTTGTTTTGCAT
chr08_9133740R	CAACAGTTTTCCAACCAAGGA
chr08_9199203F	CCTCAGGTTTCTTTGTCTGTCC
chr08_9199203R	AACATGAGCACCAATGCAAA
chr08_9203767F	CCTGCTAACCTGAAATCACTGAC
chr08_9203767R	TGGTGTTGAAGTGGAATTGG
chr08_9204810F	GGGATAAGGTTGGGAGGGTA
chr08_9204810R	CCCCCAAATAAACCCACTCT
chr08_14721279F	CTTTGCTCTTCTCCCCTTCC
chr08_14721279R	CCTGGGAGCTCGACTCTATG
chr10_15636057F	TGCTCCCGATTTAATGTGAA
chr10_15636057R	AACAGTGATGATTTGCCTCAGA
chr11_23733135F	GACTCATGGAAGGGTGCAGT
chr11_23733135R	TTTTTGACTGACTGCCCAAAT

to be continued...

Name	Sequence (5’ to 3’)
chr02_8725462F	CTGCAAGCCTTTACGTCTCC
chr02_8725462R	GGCGATGGATATGAACACTG
chr02_20049513F	ATGTTGCCCTAGCAGCAGTT
chr02_20049513R	ACTGGGGTGTCACCTATCCA
chr02_20977570F	GAGACGACCCGGAATCAG
chr02_20977570R	ATCACAAGCTCACCCCTCTC
chr02_22042282F	GCGGGGGAGTACGTGATAC
chr02_22042282R	ATGTCAGGTTGTTCGCTGGT
chr02_22043474F	ACCGGCTAAAGCTCCTCAAT
chr02_22043474R	CAATTCAAGCCCATCCCTTA
chr02_22137366F	AACGGTGTGGTCTCCTGTG
chr02_22137366R	CGGATGCCTGCAATACATATC
chr05_16018010F	TTCCCAACATGCTAACTAGTGC
chr05_16018010R	CAGTAGCCTCGTGAATGTGC
chr06_28295909F	CAAGACGGAAGGACAAGTGG
chr06_28295909R	TTGGCATGTCGAGTTGTTTT
chr06_29344094F	CATACTGGGGACAGGACGTT
chr06_29344094R	TAAGCTCTCCGCTGAACCTG
chr06_29812031F	TGCAGCTTTCTTTCCTTTGC
chr06_29812031R	GGTGGAGGACGACAACAACT
chr07_16983397F	CCGAATCCTCTTCTGCAGTC
chr07_16983397R	CTAGGGCAGTGAGCACCATT
chr07_25287466F	TCGGTTATTTGTTTGGACCAG
chr07_25287466R	AAGCCACGTCTGTTTTGGAC
chr09_4554702F	CACAAAACCCTCCCACAAAT
chr09_4554702R	GGGGAAAGGAGGAGAGAGAG
chr10_16063056F	AATGGAACGCATGACATGAA
chr10_16063056R	GCGGATCACAACGAGATTTT
chr10_16304536F	TCCGTCCACTCCCATTTCTA

to be continued...

Name	Sequence (5' to 3')
chr10_16304536R	GTCATGTGCAAAGTCGTCGT
chr10_17261561F	CTACACCAGAGCCTCCCTGA
chr10_17261561R	GCGTGTATAAATGGCAGACG
chr10_19650724F	CCAAAAATCGCCAGATCTTC
chr10_19650724R	CCCTCCCTGAACTGTCATGT
chr10_19983755F	CGATCGGCAAATCTCACC
chr10_19983755R	TGACATCTCGTCGCACTCTC
chr11_6381576F	ACCGCTGTAGCTGTGGTAGC
chr11_6381576R	TGTGGCTTGACCTTGAAGTG
chr11_16757439F	TGGACTCCAACACAAACGAA
chr11_16757439R	TGGAATTCACACACACACACA
chr11_16783119F	GTTGTTGTTGCTGCTGTTGC
chr11_16783119R	TGGATTCCTCCTCCAGTCAT
chr11_16805354F	CGGCTTATTGTGGATTTTGG
chr11_16805354R	GGGTTTCAAGTACTACTGTTTTGC
chr11_16872036F	CGATCAGATCTGGGGGAGT
chr11_16872036R	CCGAAACGAAAACCACACAT
chr11_19701863F	GCAAATCCTCGCGACTAAAT
chr11_19701863R	TTCAAGAGCTCAGCAAAATCAA

Table S2 Statistics of specific-locus amplified fragments (SLAFs).

Sample	SLAF number	Total depth	Average depth
DJ15	180,887	4,171,609	23.06×
Koshihikari	184,001	4,581,413	24.90×
Offspring	136,724	572,925	4.18×

Table S3 Statistics of different types of specific-locus amplified fragments (SLAFs).

Type	Polymorphic SLAF	Non-Polymorphic SLAF	Repetitive SLAF	Total SLAF
Number	10,812	186,083	70	196,965
Percentage	5.49	94.48	0.04	100

Table S4 Candidate genes for salt tolerance in QTL.

QTL	Gene ID	Description	Variant effects
qST1.1	Os01g0917400	CCCH-type zinc finger protein	upstream modification in DJ15 and wild rice
	Os01g0926700	Similar to secondary cell wall-related glycosyltransferase family 47	upstream modification in DJ15 and wild rice
	Os01g0926800	sec14 like protein	upstream modification, intron modification in DJ15 and wild rice
	Os01g0932500	High-affinity Potassium(K^+) Transporter 6	Ala10-Gly46del in NJ16
qST1.2	Os01g0276800	Mannose-6-phosphate receptor, binding domain containing protein.	5’ UTR modification in DJ15 and wild rice
	Os01g0279100	Subunit of magnesium-protoporphyrin IX monomethyl ester cyclase (EC:1.14.13.81), Core component of FLU-YGL8-LCAA-POR complex, Chlorophyll biosynthesis, Chloroplast development	Arg25Cys in NJ16
	Os01g0281200	Similar to Type B-like cyclin.	Ser132Arg in NJ16
	Os01g0293000	Similar to S-adenosylmethionine synthetase 1 (EC 2.5.1.6) (Methionine adenosyltransferase 1) (AdoMet synthetase 1).	3’ UTR modification in NJ16

to be continued...

QTL	Gene ID	Description	Variant effects
	Os01g0295900	Similar to cDNA clone:J023064I01, full insert sequence.	intron modification in DJ15 and wild rice
	Os01g0297700	Protein of unknown function DUF6, transmembrane domain containing protein.	5' UTR modification in DJ15 and wild rice
	Os01g0298301	Hypothetical conserved gene.	upstream modification in DJ15 and wild rice
	Os01g0298400	Myb transcription factor domain containing protein.	upstream modification in DJ15 and wild rice
	Os01g0298500	Conserved hypothetical protein.	intron modification in DJ15 and wild rice
	Os01g0299300	Hypothetical conserved gene.	intron modification in NJ16
	Os01g0302500	Knotted1-type homeobox protein OSH6.	intron modification, downstream modidification in NJ16
	Os01g0305900	Similar to R2R3 Myb transcription factor MYB-IF35.	upstream modification in DJ15 and wild rice
	Os01g0307500	Cation transporter family protein, SKC1.	upstream modification, intron modification in NJ16
	Os01g0310500	Protein kinase, catalytic domain domain containing protein.	downstream modification in NJ16
qST3	Os03g0212700	mitochondrial-processing peptidase subunit, mitochondrial precursor	His205Arg in DJ15 and wild rice
	Os03g0222700	heat shock protein DnaJ	Phe98 frameshift in DJ15 and wild rice
	Os03g0231700	squalene monooxygenase	5'UTR modification in DJ15 and wild rice
qST4.1	Os04g0583000	OsFBX149 - F-box domain containing protein	Arg179Pro in DJ15 and wild rice
	Os04g0588200	DNA2 - Putative DNA replication helicase 2 protein	His283Gln, Thr1132Ser in DJ15 and wild rice

to be continued...

QTL	Gene ID	Description	Variant effects
	Os04g0588350	Similar to nucleotidyltransferase family protein	Asp283Asn in DJ15 and wild rice
	Os04g0588700	ABC transporter family protein	Arg197Cys, Ile412Val, Phe591Leu, Thr1295Ala in DJ15 and wild rice
	Os04g0589350	OsFBX150 - F-box domain containing protein	Asn15Asp in DJ15 and wild rice
	Os04g0589600	glycosyl transferase, group 1 domain containing protein	upstream modification, intron modification in DJ15 and wild rice
	Os04g0589700	Zinc finger, RING/FYVE/PHD-type domain containing protein	upstream modification in DJ15 and wild rice
qST4.2	Os04g0461300	Similar to MIKC-type MADS-box transcription factor WM30	upstream modification in DJ15 and wild rice
	Os04g0461750	Conserved hypothetical protein	Asn179Ser in DJ15 and wild rice
	Os04g0463100	Conserved hypothetical protein	His2Asn in DJ15 and wild rice
	Os04g0468600	Heavy metal transport/detoxification protein domain containing protein	upstream modification in DJ15 and wild rice
	Os04g0471700	Similar to WRKY10 (WRKY transcription factor 35)	Phe378 duplication, upstream modification in DJ15 and wild rice
	Os04g0477300	Similar to OSIGBa0116M22.6 protein	Gln9 frameshift in DJ15 and wild rice
qST5	Os05g0339000	VHS domain containing protein	Val452Ala in DJ15 and wild rice
	Os05g0341100	Glucoamylase, putatuve domain containing protein	Val132Gly, Thr154Ala, Asp331Glu in DJ15 and wild rice

to be continued...

QTL	Gene ID	Description	Variant effects
	Os05g0341900	Similar to SAG20	Ala348Gly, Gln500Pro in DJ15 and wild rice
	Os05g0342000	Hypothetical conserved gene	Leu184Val in DJ15 and wild rice
	Os05g0342100	Wound-induced protein, Wun1 domain containing protein	Arg16 duplication, Gln80Arg in DJ15 and wild rice
	Os05g0344200	Conserved hypothetical protein	Tyr82Asp in DJ15 and wild rice
	Os05g0345700	Protein of unknown function DUF569 domain containing protein.	Cys19Arg in DJ15 and wild rice
	Os05g0346300	Similar to 40S ribosomal protein S7	Tyr64His in DJ15 and wild rice
	Os05g0346500	Glycoside hydrolase, family 85 domain containing protein	Ala16 duplication, Leu525Gln, upstream modification in DJ15 and wild rice
	Os05g0347000	TAFII55 protein conserved region domain containing protein	Ala5 frameshift in DJ15 and wild rice
	Os05g0348900	Similar to triacylglycerol lipase	Asn103Ser, Leu169Pro in DJ15 and wild rice
	Os05g0345900	Myb/SANT-like domain domain containing protein	Ser72Leu, Leu94 stop gained in DJ15 and wild rice
	Os05g0349400	Conserved hypothetical protein	Val2Ala in DJ15 and wild rice
	Os05g0353200	Hypothetical protein	Val285Glu in DJ15 and wild rice
	Os05g0353400	Armadillo-like helical domain containing protein	Phe410Leu in DJ15 and wild rice
qST6	Os06g0635700	ETHYLENE RESPONSE FACTOR 114	Arg330Leu, Val354Ala, Ile527Val in DJ15 and wild rice

to be continued...

QTL	Gene ID	Description	Variant effects
	Os06g0636100	nitrate-induced NOI protein	upstream modification, intron modification in DJ15 and wild rice
	Os06g0636600	protein kinase domain containing protein	Leu50Pro in DJ15 and wild rice
	Os06g0636700	Esterase, SGNH hydrolase-type domain containing protein	Ala57 duplication, Arg258Leu in NJ16
	Os06g0636800	Conserved hypothetical protein	Ala13-Leu16 duplication in DJ15 and wild rice
	Os06g0637800	Hypothetical gene	His14Leu, Ala32 frameshift in DJ15 and wild rice
	Os06g0639100	zinc finger, C3HC4 type domain containing protein	Leu223Pro in DJ15 and wild rice
	Os06g0639200	AP2 domain containing protein	Pro30-Ala32 deletion, Glu287Asp, Pro310Ser in DJ15 and wild rice
	Os06g0639500	protein kinase domain containing protein	Trp243Cys, Phe509Val in DJ15 and wild rice
	Os06g0640201	Hypothetical gene	Asp40His, upstream modification in DJ15 and wild rice
	Os06g0640800	Similar to Cytochrome P450 CYP71Y10	Lys146Gln, Ile223Phe in DJ15 and wild rice
	Os06t0641066	Hypothetical protein	Gly18Cys, Asp22Glu, Gln104Glu, Leu131Val in DJ15 and wild rice
	Os06g0641575	Hypothetical protein	Asp74 frameshift in DJ15 and wild rice
	Os06g0642550	Hypothetical gene	Thr59Ala, Thr467Ala in DJ15 and wild rice
	Os06g0643000	Phox-like domain containing protein	Lys422Gln in DJ15 and wild rice
	Os06g0643500	Similar to ADR11 protein	Ser24Pro in DJ15 and wild rice

to be continued...

QTL	Gene ID	Description	Variant effects
	Os06g0644600	Similar to predicted protein, WD40 repeat	Ser669Ile in DJ15 and wild rice
	Os06g0645100	Similar to F-box domain containing protein	Asn24Asp, Pro51His, Asp290Ala in DJ15 and wild rice
qST8	Os08g0437050	Hypothetical conserved gene	5'UTR modification in NJ16 and Koshihikari
	Os08g0438701	Hypothetical conserved gene	upstream modification in DJ15 and wild rice
	Os08g0453800	Serine/threonine protein kinase-related domain containing protein	upstream modification in DJ15 and wild rice
qST10	Os10g0457700	Similar to chromatin remodeling complex subunit	Ala57Val, Cys404Arg, Val421Ala, Ser1216Ala in DJ15 and wild rice
	Os10g0458600	Conserved hypothetical protein	Ser70 frameshift in DJ15 and wild rice
	Os10g0458700	Ribonuclease H domain containing protein	Asn6Asp in DJ15 and wild rice
	Os10g0458900	Exostosin-like family protein	Ser11Gly in DJ15 and wild rice
	Os10g0459300	Exostosin-like family protein	Ser225Pro, Asp493Gly in DJ15 and wild rice
	Os10g0459600	Similar to Xyloglucan galactosyltransferase KATAMARI 1 (EC 2.4.1.-) (MURUS3 protein)	Glu80Lys, ASp238Gly in DJ15 and wild rice
	Os10g0460800	Similar to H0418A01.5 protein	Gln18 stop gained in DJ15 and wild rice
	Os10g0463400	B-type response regulator, Floral inducer to promote short-day (SD) flowering pathway	Lys291Met in DJ15 and wild rice
	Os10g0464500	Zinc finger, RING-type domain containing protein	Met54Val in DJ15 and wild rice
	Os10g0464300	AWPM-19-like family protein	Arg98Gly in DJ15 and wild rice

to be continued...

QTL	Gene ID	Description	Variant effects
	Os10g0466300	Similar to Yarrowia lipolytica chromosome C of strain CLIB99 of Yarrowia lipolytica	His512Leu in DJ15 and wild rice
	Os10g0468500	Serine/threonine protein kinase-related domain containing protein	Lys216Gln in DJ15 and wild rice
	Os10g0469700	Leucine-rich repeat, typical subtype containing protein	Val39Glu, Asn172Lys, GLy482Ser in DJ15 and wild rice
	Os10g0469900	Similar to peptide transporter PTR2	Ala455Ser, Ala511Ser, Asn589Asp, Val599Ala in DJ15 and wild rice
